# Hot Embossing of Micro-Pyramids into Thermoset Thiol-Ene Film

**DOI:** 10.3390/polym12102291

**Published:** 2020-10-06

**Authors:** Dalius Jucius, Algirdas Lazauskas, Viktoras Grigaliūnas, Asta Guobienė, Linas Puodžiukynas

**Affiliations:** 1 Institute of Materials Science, Kaunas University of Technology, K. Baršausko 59, LT51423 Kaunas, Lithuania; algirdas.lazauskas@ktu.edu (A.L.); viktoras.grigaliunas@ktu.lt (V.G.); asta.guobiene@ktu.lt (A.G.); 2 Department of Physics, Kaunas University of Technology, Studentu St. 50, LT51368 Kaunas, Lithuania; linas.puodziukynas@ktu.lt

**Keywords:** hot embossing, micropatterning, thiol-ene, shape memory, thermoset, AFM

## Abstract

This paper presents the first attempt to texturize a fully crosslinked thermoset shape memory polymer using a hot embossing technique. UV-cured thiol-ene films were successfully embossed with anisotropically-etched Si (100) stamps at a temperature of 100 °C, which is about 50 °C above the glass transition temperature of the polymer. The low storage modulus of the polymer in a rubbery state allowed us to permanently emboss random micro-pyramidal patterns onto the surface of the film with high fidelity by applying 30 MPa pressure for 1 h. Atomic force microscopy (AFM) investigation showed perfect replication of the stamp micropattern with typical height of the largest inverted pyramids close to 0.7 µm and lateral dimensions in the range of 1–2 µm. Changes in surface roughness parameters of the embossed thiol-ene films after annealing them at 100 °C for 1 h or storing for 2 months in air at standard room conditions were negligible. The achieved results open new perspectives for the simple and inexpensive hot embossing technique to be applied for the micropatterning of prepolymerized thermoset shape memory films as an alternative to micropatterning using UV casting.

## 1. Introduction

Hot embossing has become a conventional method for micro- and nanostructuring of thermoplastic polymers with up to 5–10 nm resolution. It is a low-cost replication process based on the transfer of a millimeter to nanometer scale stamp pattern into the polymer at proper temperature and pressure [1,2,3]. A similar process of thermal embossing of a thin polymer film on a hard substrate, requiring a significant lateral flow for complete filling of the stamp cavities, is known as nanoimprint lithography (NIL) [4,5,6]. Scale-up development of hot embossing for large-area and high-throughput patterning led to the introduction of very an effective roll-to-roll imprint process [7,8]. Numerous applications of hot embossing include holography, optical waveguides, organic light-emitting diodes, nanophotonic metamaterials, thin film solar cells, microfluidics, biochips and so on [1,9,10,11].

Recently, hot embossing has also been applied to form micro- and nanostructures on the surface of shape memory polymers (SMPs). The shape memory effect on micro- and nanoscale is very interesting as it has tremendous potential to be used to fabricate dynamic surface topologies yielding “switchable” surfaces that are able to respond to specific external stimuli with considerable changes in their properties [12]. However, this effect is still underexplored. SMP studies on micro- and nanoscale started only in 2004 when the first SMP laser heating and atomic force microscopy (AFM) tip indentation tests were performed [13,14]. So far, only thermoplastic elastomer type SMPs have been studied in more detail. The by far most popular was Tecoflex amorphous elastomer, which was patterned with the aim to create surfaces with switchable adhesion [15], to investigate recovery of micro- and nanostructures [16,17], to produce diffractive optical elements [18] and disordered gratings [19], to fabricate microstructured self-demolding molds [20]‚ etc. Other examples of thermoplastic SMP texturing involve embossing of microprismatic patterns on polyvinyl acetate films, fabrication of micro-optical components via compression molding of poly(ethylene-co-vinyl acetate), microprism/microlens embossing on the surface of polyvinyl acetate/polyurethane multilayer films and nanoimprint of the permanent micro- and nanometer-scale surface textures into Nafion film [21,22,23,24].

On the other hand, micro- and nanostructuring of thermoset type SMPs is just taking its first steps. Only a few studies on this topic related to heat polymerization on the mold or hot embossing of the partially crosslinked SMP and subsequent crosslinking at elevated temperature can be found in the scientific literature, including preparation of superhydrophobic surfaces by curing epoxy-based material on a micro/nanostructured substrate [25,26], tuning wettability by using surface patterned crosslinked polycyclooctene [27], formation of photonic structures in polydiolcitrates (PDCs) [28] and thermal embossing of micropillars and subsequent thermal crosslinking of poly(e-caprolactone) with allyl alcohol and Fe_3_O_4_ nanoparticles [29]. Thermosets capable of forming covalently crosslinked networks are usually tougher than thermoplastic polymers and demonstrate high shape fixity and shape recovery rates and thermal stability. Thus, they are of great interest for the control of topography-dependent material properties, switchable carriers of information and deployable aerospace structures [30]. Click polymerizations, such as Diels-Alder, thiol-ene and azide-alkyne cycloaddition reactions, have been successfully employed for the synthesis of various functional polymers and are also promising for the production of shape memory thermosets [31]. Thiol-ene polymer systems are of particular interest as they present numerous advantages including fast polymerization, low volume shrinkage, homogeneity of the polymer network, flexibility and high optical transparency of the polymerized films [32,33]. It was reported that thiol-ene polymer networks exhibit a good shape recovery and self-healing effect [34]. Recently, our group demonstrated an efficient recovery of microstructured thiol-ene films fabricated via casting and a UV polymerization technique on silicon substrates textured using electron beam lithography [35].

This paper presents the first attempt to texturize a fully crosslinked thermoset shape memory polymer using a hot embossing technique. Embossing stamps were fabricated via simple maskless texturization of a silicon surface. We have succeeded in embossing micro-pyramidal patterns of the stamps on the surface of UV-cured thiol-ene film above the glass transition temperature of the polymer. Embossed micropatterns were characterized using optical and atomic force microscopy. Annealing and aging effects on the embossed texture were studied as well.

## 2. Materials and Methods

1,3,5-triallyl-1,3,5-triazine-2,4,6(1H,3H,5H)-trione (TTT, trifunctional allyl component), pentaerythritol tetrakis(3-mercaptopropionate) (PETMP, tetrafunctional thiol component), 2,2-dimethoxy-2-phenylacetophenone (DMPA, photoinitiator) and potassium hydroxide (KOH) were purchased from Sigma-Aldrich (St. Louis, MO, USA). All reagents were used without further purification. Double side polished silicon (100) wafers with a thickness of 500 ± 20 µm were supplied by University Wafer (Boston, MA, USA). Fused silica wafers with a thickness of 500 µm and an average roughness of wafer surface R_a_ < 1 nm were supplied by Siegert Wafer (Aachen, Germany).

Stamps for hot embossing were fabricated using simple maskless texturization via anisotropic etching of (100) silicon in aqueous KOH solution. Si wafers were scribed and cleaved into rectangular pieces of 10 by 10 mm. These pieces of Si were cleaned ultrasonically in acetone and ethyl alcohol and then placed into the bath with 30 wt% aqueous solution of KOH. With the aim to determine the optimal etching conditions for the fabrication of pyramid-like stamp features, the etching time was varied from 2 to 10 min and the etching temperature was varied from 50 to 80 °C. Etched Si stamps were rinsed in deionized water for 2 min and dried with nitrogen flow.

**Scheme 1:**
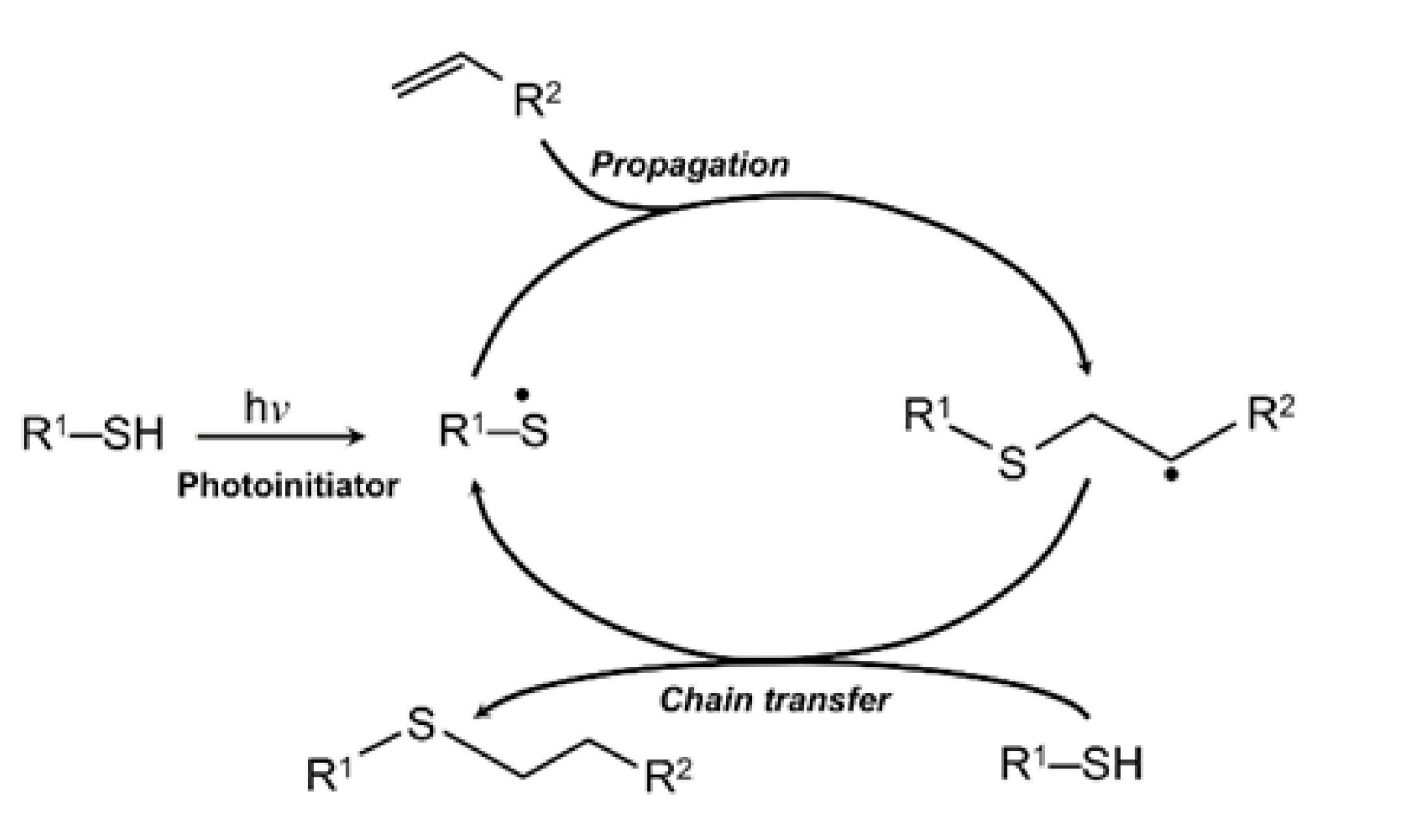
The photochemical radical-initiated step-growth mechanism of the thiol-ene polymerization reaction, reproduced from [36], with permission from © The Royal Society of Chemistry.

Photopolymerizable thiol-ene composition was prepared as a mixture of PETMP and TTT with 1:1 stoichiometric ratio of thiol to ene functional groups, containing 1 wt% of DMPA. Details of the preparation procedure have been reported previously [34]. The fused silica wafers were cleaned ultrasonically in acetone and isopropyl alcohol to remove any dirt from the surface and dried in air before use. The clear, colorless, viscous mixture of PETMP and TTT was applied on the fused silica wafers as a 100 µm-thick layer via the Meyer rod coating method. All the samples were UV cured simultaneously at the intensities of 1.64 mW/cm^2^ (254 nm wavelength) and 0.8 mW/cm^2^ (365 nm wavelength) for 2 min. The photochemical radical-initiated step-growth mechanism of the thiol-ene polymerization reaction is presented in Scheme 1. After that, cured PETMP-TTT films were gently peeled from the fused silica substrates, cut into pieces of 15 × 15 mm and used for the hot embossing experiments.

**Figure 1:**
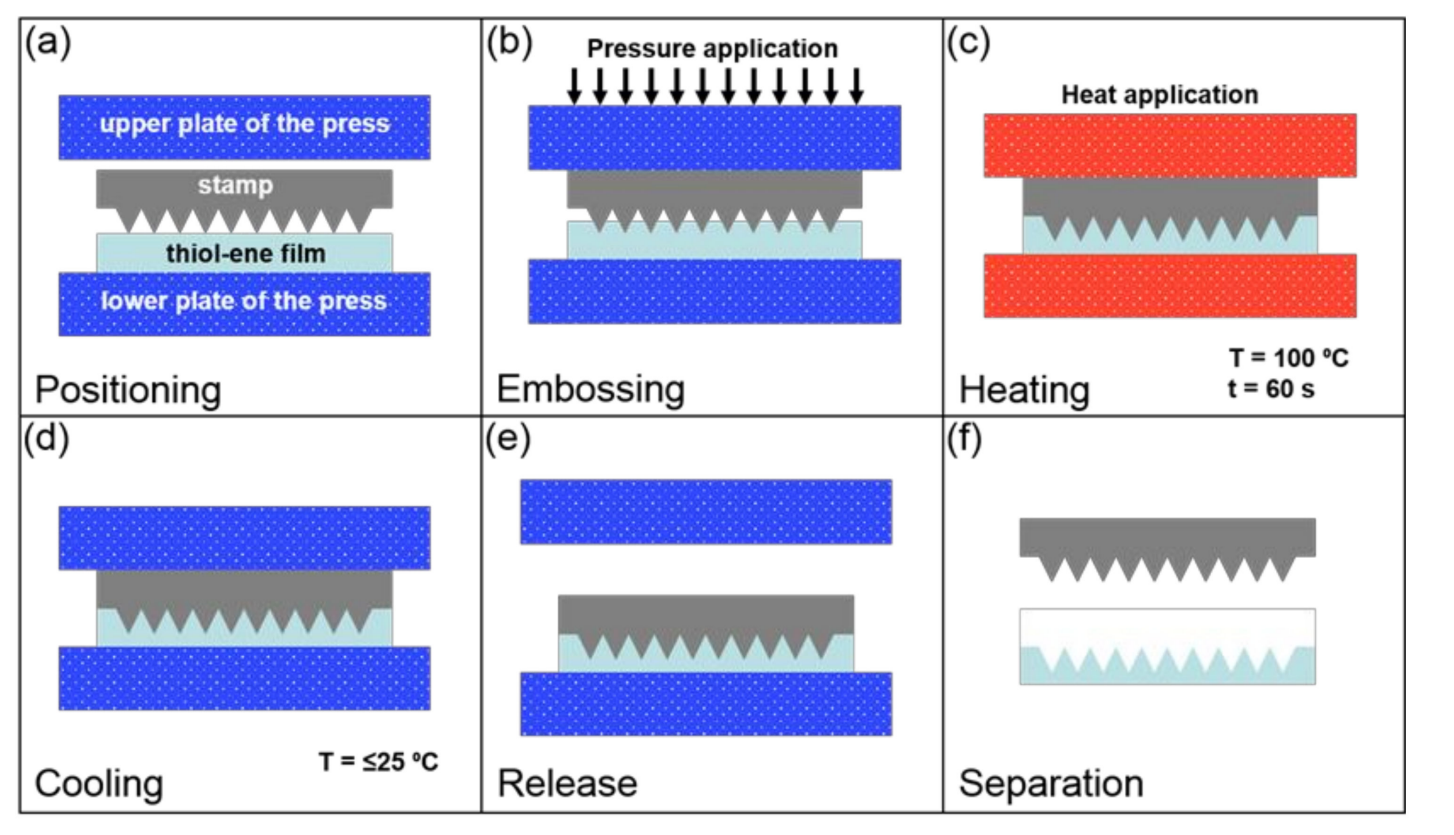
Main steps of the hot embossing process.

Hot embossing was performed using electrically heated laboratory press. Thiol-ene film brought into contact with a Si imprint stamp was positioned between cool press plates, and pressure ranging from 10 to 30 MPa was applied. The temperature was raised to 100 °C and kept constant for 1 h. Then, the heating was turned off and natural cooling of the press took place. At 25 °C, pressure was released and the stamp was manually separated from the polymer film. The main steps of the hot embossing process are presented in Figure 1. A cold imprint was carried out with the same press at room temperature using 30 MPa pressure for 70 h.

Annealing of the embossed films was carried out on a POLOS Hotplate 200S (APT GmbH, Bienenbüttel, Germany) at 100 °C for 1 h. The precision digital temperature controller of the hotplate enabled adjustable temperature steps of 1 °C. Differences in temperature of the hot surface did not exceed ± 0.5 °C. The aging effects of the hot embossed thiol-ene films were studied after two months of sample storage in air at standard room conditions.

A Raith e-LiNE Plus scanning electron microscope (SEM) integrated with an electron beam lithography system (Raith GmbH, Dortmund, Germany) was used to observe the fabricated Si imprint stamps.

The thermomechanical properties of the thiol-ene films were determined via dynamic mechanical analysis (DMA). DMA was carried out in a tension mode using a MCR302 rheometer with DMA accessories (Anton Paar GmbH, Graz, Austria). Rectangular samples with dimensions 15 mm × 5 mm × 0.1 mm were cut for the tests from the free-standing thiol-ene films. The samples were strained at a rate of 1 Hz and to a total 0.05% strain while heating 2 °C/min. The storage modulus and loss tangent (tan δ) were recorded as functions of temperature from 26 to 100 °C. Average values from the testing of six samples are presented and analyzed in this paper.

Imaging of the films’ surface was done using an optical microscope OPTIKA B-600 MET (Optika Srl, Ponteranica, Italy) with magnification of 750×. Optical microscope images were captured using a 2560 × 1920 pixel digital camera Optikam Pro 5LT.

Surface morphology of the thiol-ene films was investigated using atomic force microscopy (AFM). AFM experiments were carried out at room temperature using a NanoWizardIII atomic force microscope (JPK Instruments, Bruker Nano GmbH, Berlin, Germany). AFM images were collected using a V-shaped silicon cantilever (spring constant of 3 N/m, tip curvature radius of 10.0 nm and cone angle of 20°) operating in contact mode. The data were analyzed using JPKSPM Data Processing software (Version spm-4.3.13, JPK Instruments, Bruker Nano GmbH) and open source software Gwyddion 2.53, which is supported by the Czech Metrology Institute and freely available at http://www.gwyddion.net. Surface roughness parameters of the films were calculated by averaging results obtained at three different positions on each of the four identical samples.

## 3. Results and Discussion

**Figure 2:**
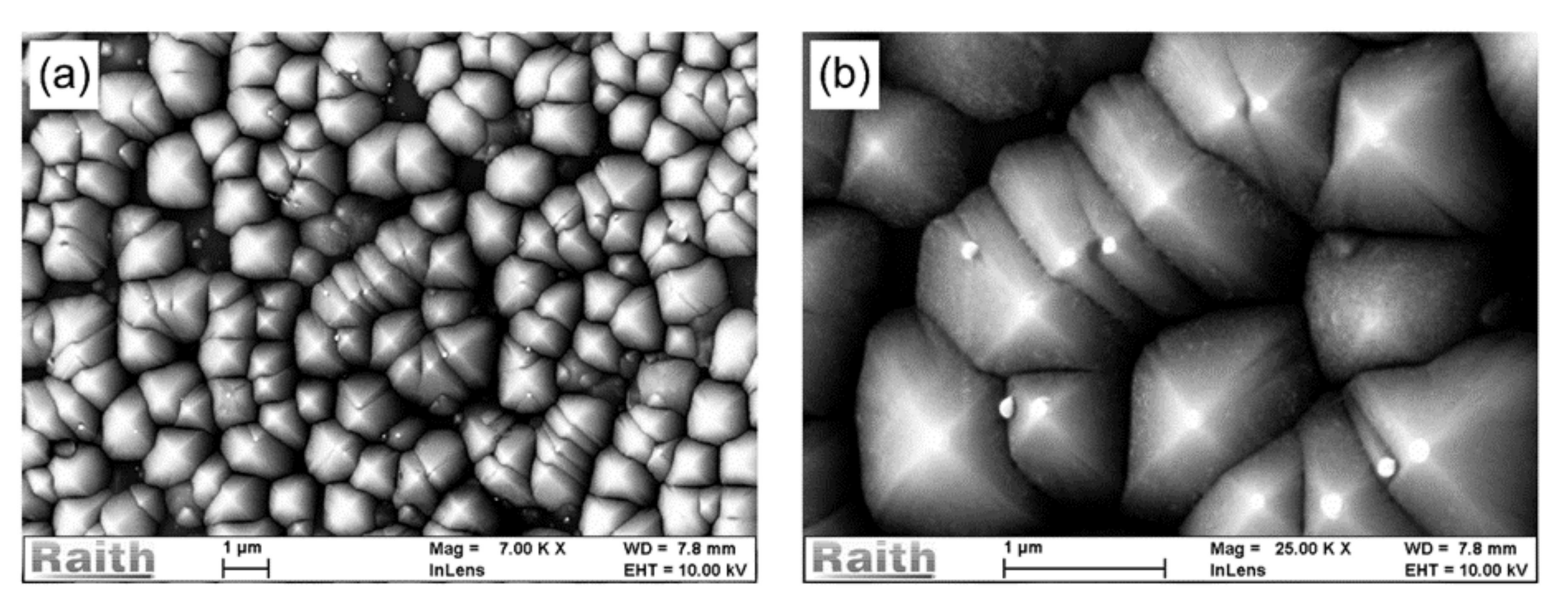
Scanning electron microscope (SEM) micrographs of the silicon surface etched in a 30 wt% KOH aqueous solution at 65 °C for 10 min. Magnification: (**a**) 7000× and (**b**) 25000×.

It is well known that pyramid-like structures of various sizes with (111) sidewall can be created on Si (100) substrate using anisotropic etching in aqueous KOH solution, as the etching rate in (111) direction is very slow [37]. The etching rate of silicon (100) is mainly dependent on the concentration and temperature of the etching solution [38]. We tried to fabricate the stamps for hot embossing using 30 wt% aqueous KOH solution and varying the etching temperature from 50 to 80 °C. The etching time was varied from 2 to 10 min and the optimal etching conditions for the fabrication of the pyramid-like stamp features (resulting in a homogeneous etching and black color of the etched stamp surface) were determined employing an optical microscope and SEM. Optical microscope images of silicon stamps prepared under different etching conditions are presented in Table S1, Supplementary Materials. The experimental results showed that an etching time of 2 to 6 min was insufficient and 10 min etching at 80 °C resulted in over-etching of the Si surface. The best results were obtained by 10 min etching at 65 °C (Figure 2). Etching at these conditions resulted in a homogeneous black surface of the stamp. The whole Si surface was covered with sharp rectangular micro-pyramids of relatively uniform size and density. Randomly distributed pyramids with clearly defined smooth facets and typical lateral dimensions of 0.5–2 µm were observed. Due to the dense arrangement, some of them were aggregated and partially merged with each other. The tiny particles seen on the pyramids can be attributed to minor sample contamination. The final texture of the etched stamps was quite deep and suitable for hot embossing experiments.

**Figure 3:**
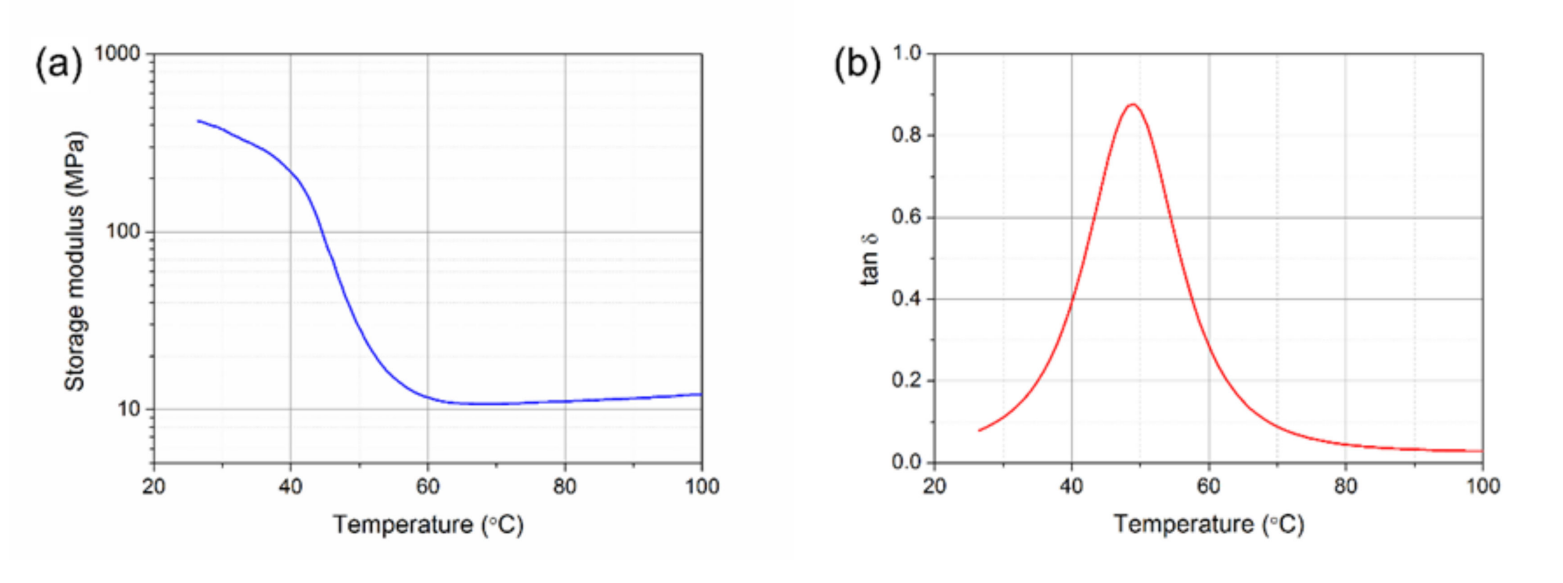
(**a**) Storage modulus and (**b**) damping factor (tan δ) as a function of temperature for the UV-casted thiol-ene films.

Thermomechanical properties of the UV casted thiol-ene films were determined from dynamic mechanical analysis (DMA). Temperature dependence of the storage modulus and damping factor is presented in Figure 3. This dependence is characteristic for the modulus and tan δ in an amorphous polymer network. The storage modulus of the films (Figure 3a), representing the energy stored in the elastic structure of the material, at room temperature is rather high (420 MPa at 26  °C) and decreases slowly with the increase of temperature at glassy state. Rapid drop in the modulus associated with the glass transition starts at about 42 °C and ends at around 55 °C. In the rubbery plateau region from 60 to 100 °C, the storage modulus does not exceed 11–12 MPa. The curve of the damping factor (Figure 3b), indicating the relative degree of energy dissipation at different temperatures, is narrow and symmetric, thus it confirms the high homogeneity of the tested thiol-ene films. The glass transition temperature (*T_g_*) of the films was determined to be the temperature of the damping factor curve maximum equal to 48.5 °C. The width of the glass transition region (*ΔT_g_*), determined as the full width at half-maximum of the tan δ curve, is equal to 16 °C. It is evident that *ΔT_g_* is much narrower than for the more heterogeneous UV polymerizable methacrylate networks [39]. This indicates a very uniform crosslink density and highly desirable mechanical behavior of the UV-casted thiol-ene films.

The crosslink density of the films was calculated using an expression deduced from Flory’s rubber elasticity theory [40]:

(1) $$v_{e}=\frac{E_{r}}{{3RT}_{g}}\;$$

where *v_e_* (mol m^−3^) is the molar concentration of crosslinks, *E_r_* (Pa) is the rubbery modulus obtained from the storage modulus curve at 50 °C above *T_g_*, *R* = 8.314 J K^−1^ mol^−1^ is the universal gas constant, and *T_g_* (K) is the glass transition temperature of the polymer obtained from the tan δ curve. Equation (1) is valid only for the polymers with a rubbery modulus in the range of 2 to 200 MPa. In our case, the rubbery modulus of the films was equal to 12.2 MPa, and we could apply the kinetic rubber elasticity theory without restrictions. Calculations have resulted in a rather high value of the crosslink density (*v_e_* = 1516 mol m^−3^), which is almost twice higher than reported by other authors for the photochemically-cured thiol-ene films [41,42].

The shape memory of analogous, UV-casted thiol-ene films was recently tested by our group and reported in [35]. Thus, in this study additional shape memory tests were not performed.

Surface morphology of the UV-casted thiol-ene films was evaluated using AFM. The area roughness parameters such as root mean square (RMS) roughness *S_q_*, skewness *S_sk_*, excess kurtosis *S_ku_* and maximum peak to valley height *S_z_* were calculated to quantify the embossed micropatterns. The maximum peak to valley height *S_z_* indicates the absolute vertical distance between the maximum peak height and the maximum valley depth in the analyzed area. The other parameters are computed from the central moments of *i*-th order *μ_i_*, i.e., the moments of a probability distribution of a height about its mean value:

(2) $$\mu_{i}=\frac{1}{N}\overset{N}{\underset{n=1}{\sum }}{\left(z_{n}-\bar{z}\right)}^{i}$$

where *N* is the total number of data points, *z_n_* is the height at position *n*, and $\bar{z}$ is the mean height. *S_q_*, *S_sk_* and *S_ku_* are expressed by the following formulas:

(3) $$S_{q}=\sqrt{\mu_{2}\;},$$

(4) $$S_{sk}=\frac{\mu_{3}}{\sqrt{\mu_{2}^{3}}},$$

(5) $$S_{ku}=\frac{\mu_{4}}{\mu_{2}^{2}}-3$$

Thus, *S_q_* is a root mean square average of the height deviations taken from the mean height plane, *S_sk_* is a measure of the asymmetry of the height probability distribution about the mean height and *S_ku_* is a measure of the “tailedness” of the height probability distribution.

**Figure 4:**
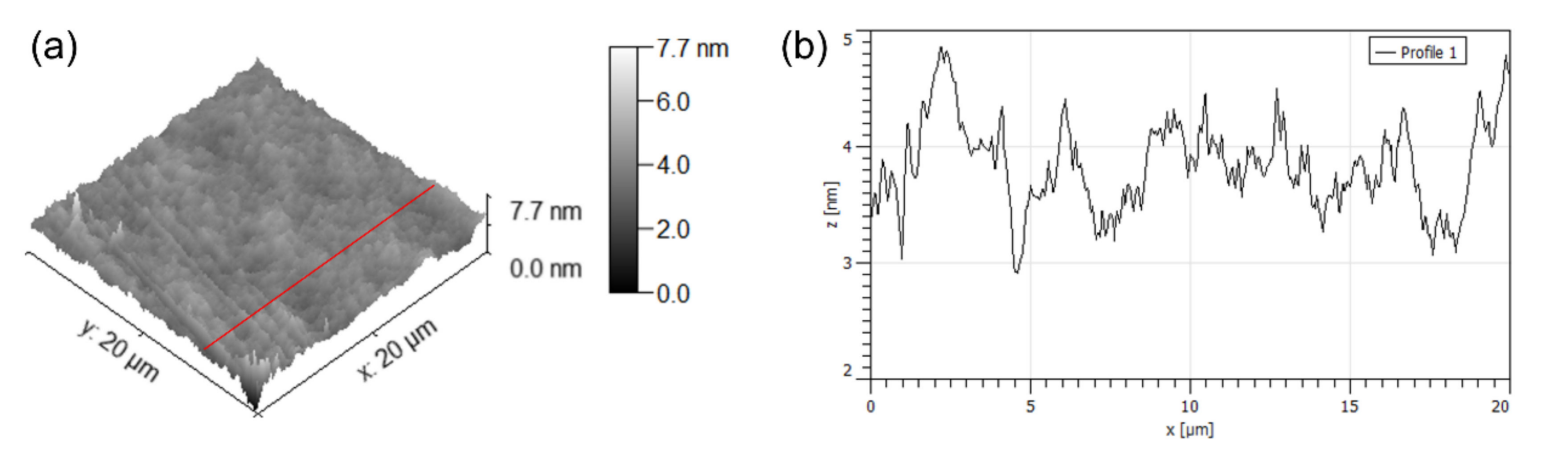
(**a**) Three-dimensional atomic force microscopy (AFM) image and (**b**) profile of the free-standing, UV-casted thiol-ene film. The red line indicates the location where the AFM profile was extracted.

AFM scans revealed quite a smooth surface having an average RMS roughness *S_q_* = 386 pm and symmetric leptokurtic height distribution with *S_sk_* = 0.06 and *S_ku_* = 3.88. Figure 4 shows a characteristic 3D AFM topographical image and profile of the photopolymerized thiol-ene film.

Imprint conditions for the hot embossing of the UV-casted thiol-ene films were selected based on the DMA results. Hot embossing was performed at a temperature of 100 °C, which is about 50 °C above the *T_g_* of the polymer. The imprint pressure was varied from 10 MPa, which is just below the thiol-ene storage modulus in the rubbery plateau, to 30 MPa, exceeding the storage modulus almost threefold.

**Figure 5:**
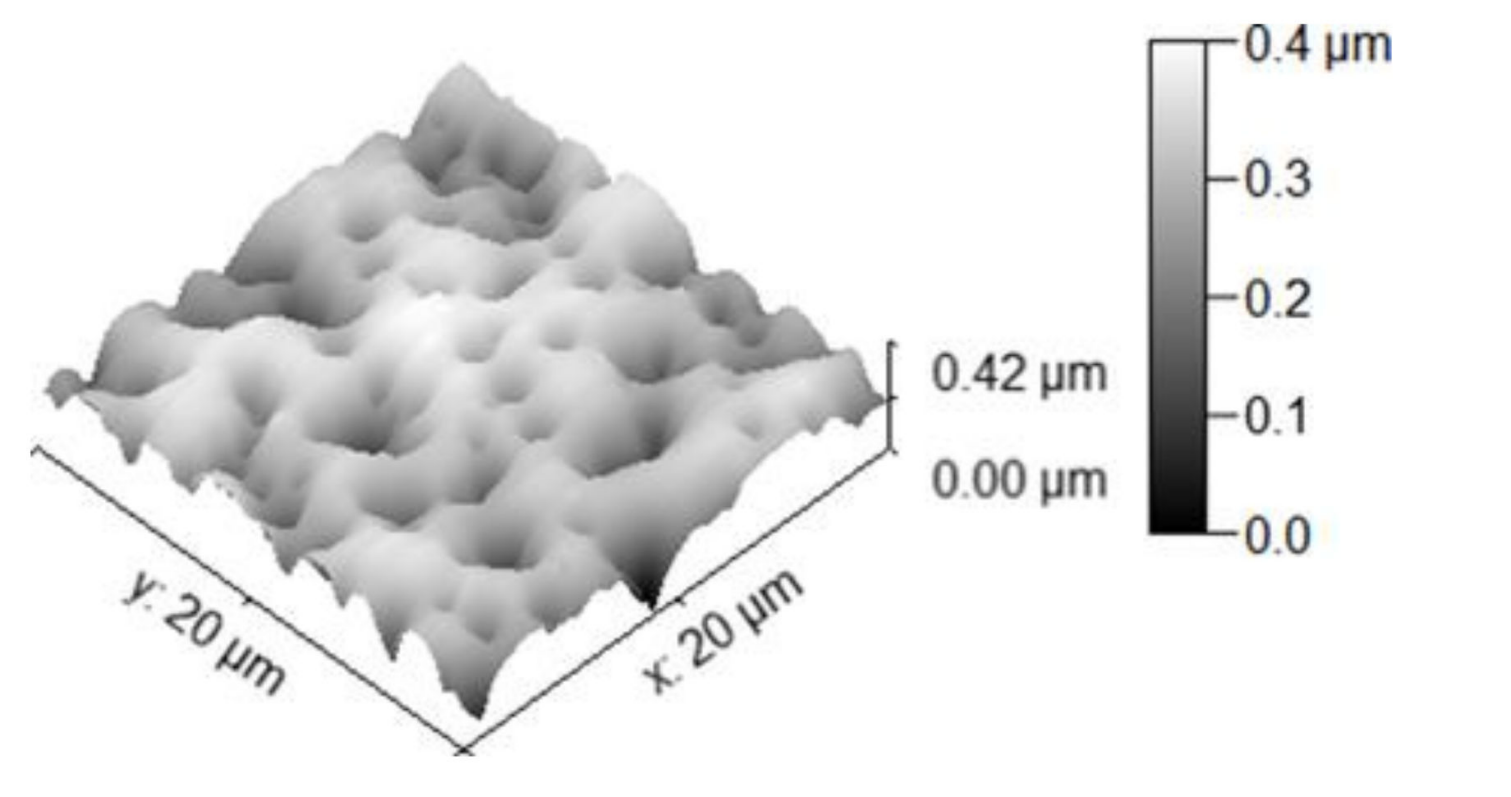
Three-dimensional AFM image of the thiol-ene surface hot embossed at 100 °C under 10 MPa.

Hot embossing of the textured Si stamp performed at a temperature of 100 °C under the pressure of 10 MPa for 1 h resulted in incomplete replication of the stamp features (Figure 5). Only vertices of the largest micro-pyramids were successfully embossed into the thiol-ene surface due to the higher instantaneous pressure at the initial stages of the imprint process. Further imprinting led to an increase in contact area and reduction of the instantaneous pressure below the thiol-ene storage modulus, preventing replication of the depressed stamp features.

**Figure 6:**
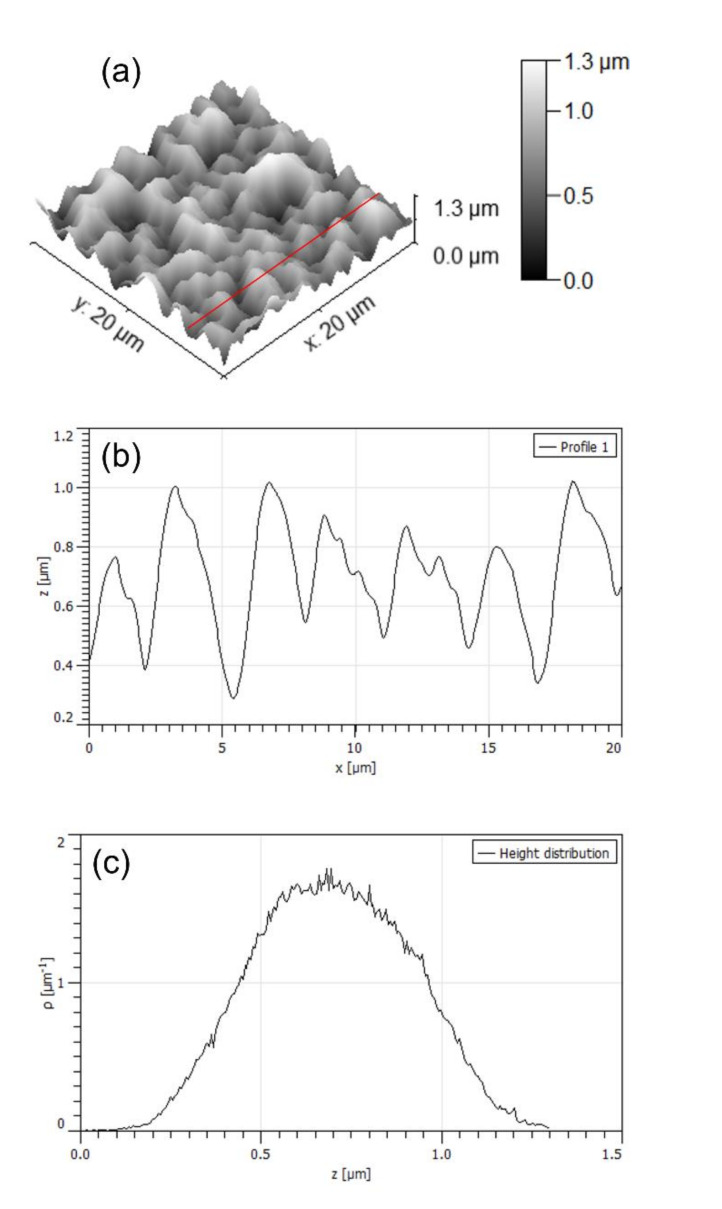
(**a**) Three-dimensional AFM image, (**b**) profile and (**c**) height distribution of the thiol-ene surface hot embossed at 100 °C under 30 MPa for 1 h. The red line indicates the location where the AFM profile was extracted.

An increase in imprint pressure of up to 30 MPa resulted in a perfect replication of the stamp microtexture (Figure 6). The maximum peak to valley height *S_z_* for the 20 µm × 20 µm scans of the replicated surfaces exceeded 1.3 µm, whereas the typical height of the largest inverted pyramids was close to 0.7 µm. The average RMS roughness of the replicas was equal to 213 nm. The height distribution of the embossed surfaces was nearly normal and slightly asymmetric with *S_sk_* = 0.19 and *S_ku_* = − 0.52.

**Figure 7:**
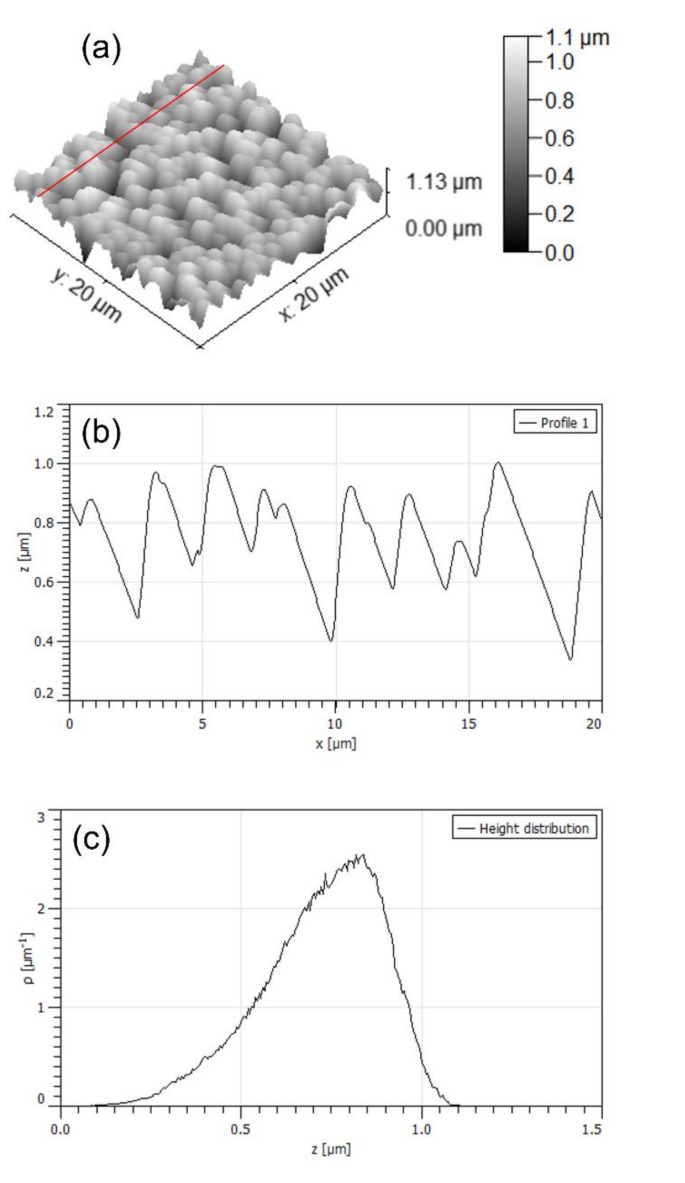
(**a**) Three-dimensional AFM image, (**b**) profile and (**c**) height distribution of the Si imprint stamp. The red line indicates the location where the AFM profile was extracted.

For comparison, we also performed an AFM analysis of the stamp surface. A characteristic AFM image, profile and height distribution of the Si imprint stamp are presented in Figure 7. Evidently, the 3D AFM topographical image of the stamp is in a good agreement with the SEM images shown in Figure 1. The determined typical height of the largest Si micro-pyramids was in between 0.6 µm and 0.7 µm. The average RMS roughness of the stamp was equal to 187 nm. The height distribution of the stamp surface was close to normal, but negatively skewed, with *S_sk_* = − 0.61 and *S_ku_* = 0.05. The negative skewness can be associated with the rare deep pits in the areas of the stamp surface where no micro-pyramids are formed. The highest protrusions of the replicas, corresponding to the deepest pits of the stamps, are the cause of the positive skewness of the embossed surfaces. Change of the excess kurtosis from 0.05 for the stamp to − 0.52 for the replica could be explained by some rounding and decrease in size of the infrequent extreme spikes most distant from the surface mean plane. It is interesting to note that the maximum peak to valley height of the replicas was even increased compared to the stamp. Observed changes in *S_z_* were possibly caused by an imparted microwaviness of the textured thiol-ene films after embossing at high pressure.

**Figure 8:**
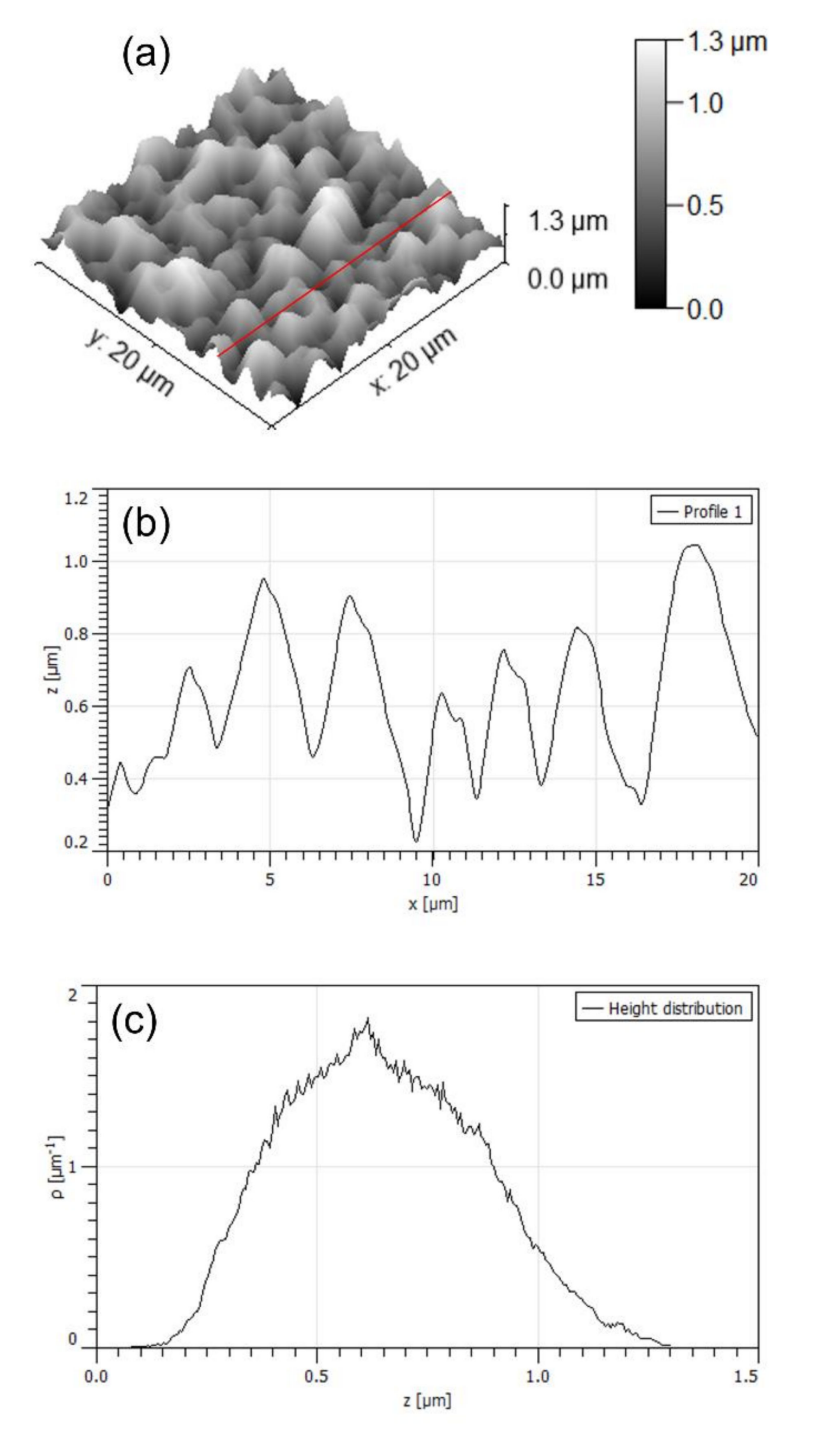
(**a**) Three-dimensional AFM image, (**b**) profile and (**c**) height distribution of the thiol-ene surface hot embossed at 100 °C under 30 MPa and annealed at 100 °C for 1 h. The red line indicates the location where the AFM profile was extracted.

**Figure 9:**
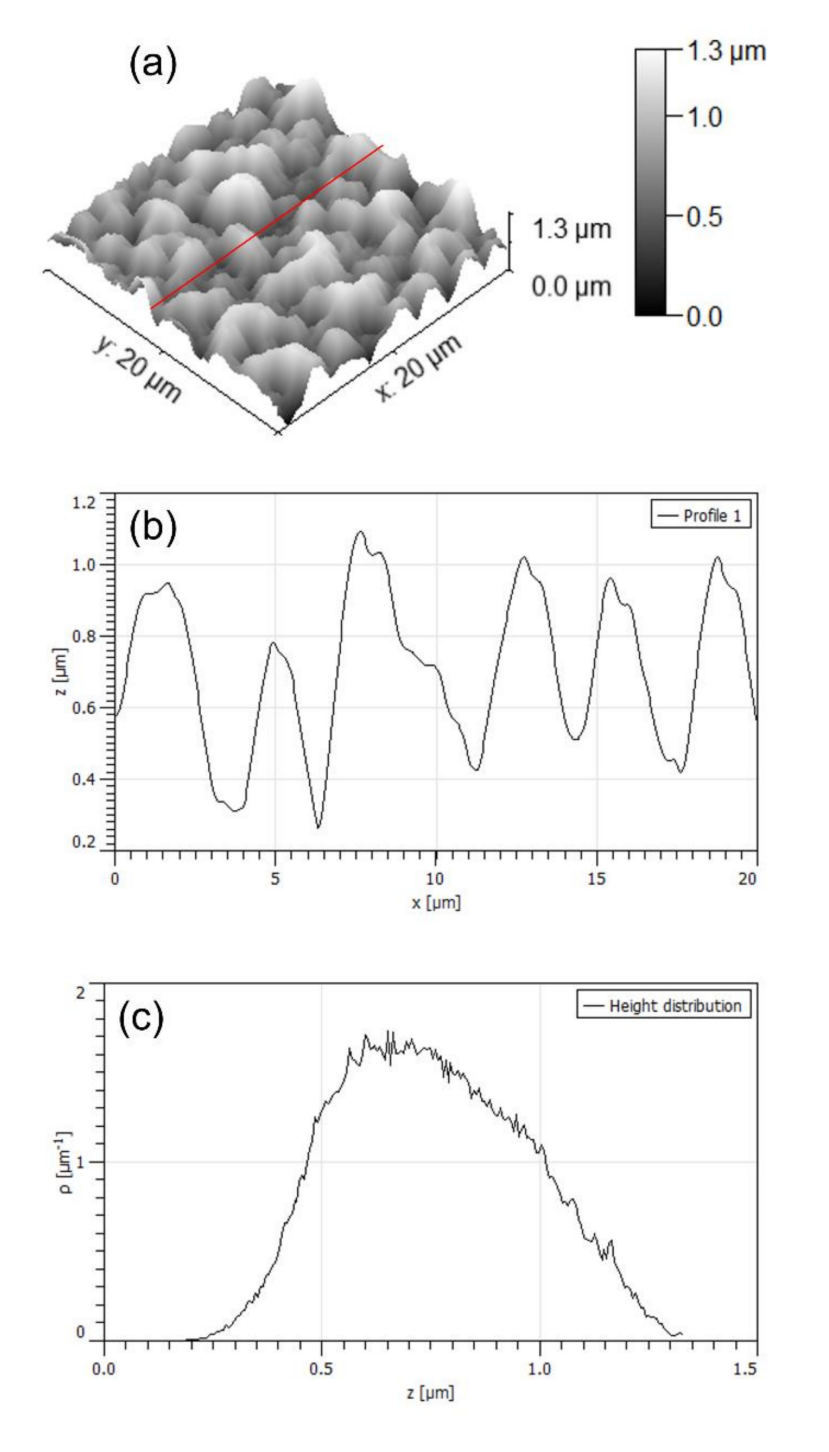
(**a**) Three-dimensional AFM image, (**b**) profile and (**c**) height distribution of the thiol-ene surface hot embossed at 100 °C under 30 MPa and aged for two months in air at standard room conditions. The red line indicates the location where the AFM profile was extracted.

Stability of the patterns embossed at 100 °C under 30 MPa was studied by annealing some of the imprinted thiol-ene films on the hotplate at 100 °C for 1 h and by storing the others for two months in air at standard room conditions. Characteristic AFM images, profiles and height distributions of the annealed and aged films are presented in Figure 8 and Figure 9, respectively. It is evident that embossed patterns were quite stable at high temperatures as well as over time. The average roughness parameters of the annealed films were as follows: *S_q_* = 218 nm, *S_sk_* = 0.22 and *S_ku_* = − 0.53. Identical parameters of the aged films were nearly the same: *S_q_* = 215 nm, *S_sk_* = 0.19 and *S_ku_* = − 0.59. Thus, compared to the just embossed thiol-ene films, changes in the surface roughness parameters were negligible.

**Figure 10:**
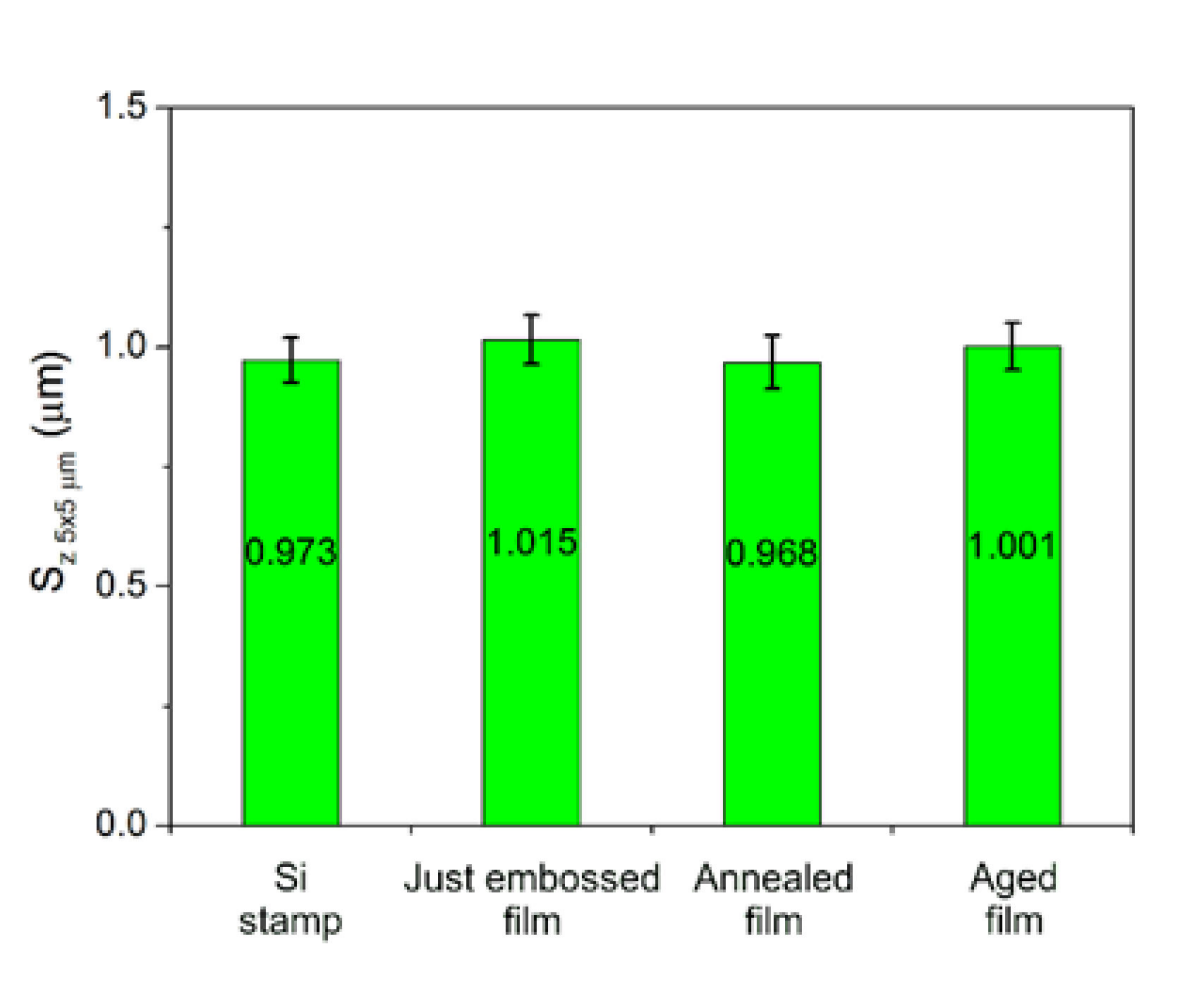
Maximum peak to valley heights in 5 µm × 5 µm areas of the Si stamps and textured thiol-ene films embossed at 100 °C under 30 MPa for 1 h.

The average *S_z_* for the 20 µm × 20 µm scans of the Si stamps and the just embossed under 30 MPa, annealed and aged microtextured thiol-ene films was equal to 1.134, 1.301, 1.305 and 1.314 µm, respectively. Thus, the estimated value of *S_z_* for the just embossed replicas was 14.7% higher compared to the stamp. Influence of subsequent annealing or aging on *S_z_* was insignificant. To examine our hypothesis about the influence of the imparted macrowaviness on *S_z_* of the embossed thiol-ene films, all 20 µm × 20 µm AFM scans were divided into 16 rectangular areas with dimensions of 5 µm × 5 µm. Each area was large enough to contain at least one high protrusion of the tested surface. Local maximum heights for all 5 µm × 5 µm areas were determined and averaged over a particular scan and all the scans of identical surfaces. The resulting averaged maximum peak to valley heights *S_z_*_5 × 5 µm_ together with error bars representing the standard deviation are presented in Figure 10. It can be seen that differences in the averaged maximum height *S_z_*_5 × 5 µm_ of the stamps and the hot embossed thiol-ene films are small and do not exceed the standard deviation. Thus, dividing of the AFM scans into 5 µm × 5 µm areas was very useful in minimizing the influence of the film macrowaviness and allowed a more precise assessment of the maximum replica height.

**Figure 11:**
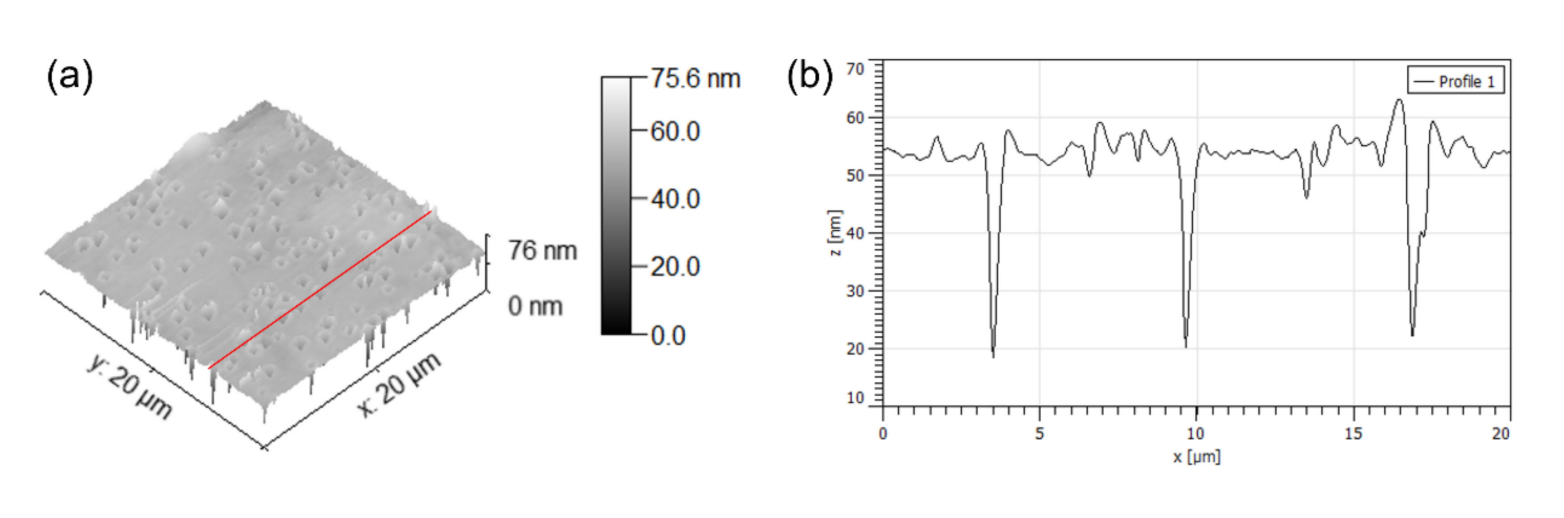
(**a**) Three-dimensional AFM image and (**b**) profile of the thiol-ene surface imprinted at room temperature under 30 MPa for 70 h. The red line indicates the location where the AFM profile was extracted.

**Figure 12:**
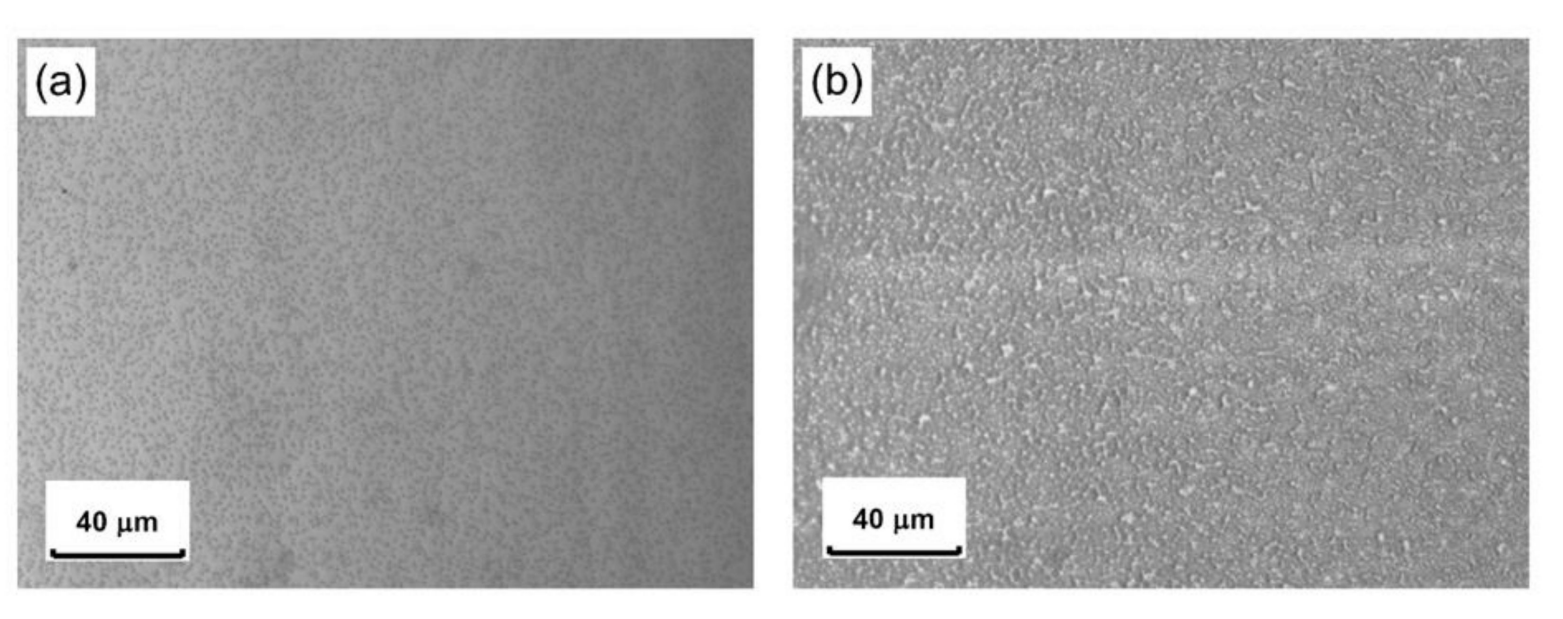
Optical microscope images of the thiol-ene surface at 750x magnification: (**a**) imprinted at room temperature under 30 MPa for 70 h, (**b**) hot embossed at 100 °C under 30 MPa for 1 h.

Finally, with the aim to investigate the imprintability of the surface below the glass transition temperature of the polymer, we tried to emboss the thiol-ene film at room temperature. The imprint of the Si stamp was performed under the pressure of 30 MPa for 70 h. In this case, despite the long imprint time, only the vertices of the highest Si micro-pyramids were embossed into the polymer (Figure 11), resulting in an asymmetric thiol-ene surface with 30–40 nm negative spikes. The rest of the surface of the film remained nearly unchanged as the storage modulus of the thiol-ene at room temperature was tenfold higher than the applied imprint pressure. On the other hand, optical microscope investigation revealed that our imprint setup was able to ensure high uniformity of the imprints over the entire surface of the stamp in the case of both the room temperature and hot embossing (Figure 12). The bright spots seen in Figure 12b represent the bases of inverted pyramids embossed into thiol-ene film at 100 °C. Meanwhile, embossing at the room temperature results in incomplete transfer of the stamp features and we can only see embossed tops of the pyramids as darker, needle prick-like areas on the smooth grey surface of the film in Figure 12a. Quantitative analysis of the imprinted surfaces was performed using AFM. To evaluate the uniformity of the imprints, we measured the maximum peak to valley height *S_z_* for the 20 µm × 20 µm scans at three different positions on each of the four identical samples. AFM measurements revealed that, at a confidence level of 95%, the maximum height *S_z_* for the surfaces imprinted at room temperature under 30 MPa for 70 h and for the surfaces hot embossed at 100 °C under 30 MPa for 1 h was 72.51 ± 3.52 nm and 1.301 ± 0.027 µm, respectively. Thus, the *S_z_* confidence interval for the imprints made at room temperature and at 100 °C was equal to ± 4.9% and ± 2.1%, respectively. 

## 4. Conclusions

This study for the first time demonstrated the applicability of the hot embossing technique for the texturing of crosslinked thermoset shape memory polymer films.

UV-cured thiol-ene films were successfully embossed with anisotropically-etched Si (100) stamps at a temperature of 100 °C, which is about 50 °C above the glass transition temperature of the polymer. At this temperature the amorphous polymer network of the thiol-ene with a calculated crosslink density of *v_e_* = 1516 mol m^−3^ gained enough energy to be transformed from a rigid glassy state with a storage modulus of 420 MPa to a significantly more flexible rubbery state with a storage modulus of only 11–12 MPa. The low storage modulus of the polymer in a rubbery state allowed us to permanently emboss random micro-pyramidal patterns onto the surface of the film with high fidelity by applying 30 MPa pressure for 1 h.

AFM investigation showed perfect replication of the stamp micropattern with typical height of the largest inverted pyramids close to 0.7 µm and lateral dimensions in the range of 1–2 µm. The observed 14.7 % increase in the maximum peak to valley height for the 20 µm × 20 µm scans of the replicas compared to the stamp was associated with the imparted macrowaviness of the embossed films and effectively reduced to the limits of standard deviation by calculating the averaged local maximum heights for the 5 µm × 5 µm areas.

Changes in surface roughness parameters of the embossed thiol-ene films after annealing them at 100 °C for 1 h or storing for 2 months in air at standard room conditions were negligible. Embossed patterns were quite stable at high temperatures as well as over time. The observed slight decrease in the averaged local maximum height after the annealing and aging can be related to the processes of polymer relaxation.

The achieved results open new perspectives for the simple and inexpensive hot embossing technique to be applied for the micropatterning of prepolymerized thermoset shape memory films as an alternative to micropatterning using UV casting. Such microembossed films could be useful for a wide range of applications including manipulation of the surface wettability, shape memory holograms and diffractive optical elements, switchable optical devices, etc. The main advantages of microstructured shape memory thermosets over thermoplastics would be their high shape fixity and thermal stability.

## Supplementary Materials

The following are available online at https://www.mdpi.com/2073-4360/12/10/2291/s1, Table S1: Anisotropic etching of Si (100) in 30 wt% aqueous KOH solution.
