# Supplementary material for: Hot Embossing of Micro-Pyramids into Thermoset Thiol-Ene Film
Source: Polymers (Basel). 2020 Oct 6;12(10):2291. doi: 10.3390/polym12102291 (PMC7600293; doi:10.3390/polym12102291)
Supplement: Supplementary file 1 [file polymers-12-02291-s001.pdf]

polymers

MDPI

Supplementary Materials

Hot Embossing of Micro-Pyramids into Thermoset Thiol-Ene Film

Dalius Jucius [1,*], Algirdas Lazauskas [1], Viktoras Grigaliūnas [1], Asta Guobienė [1] and Linas Puodžiukynas [2]

[1] Institute of Materials Science, Kaunas University of Technology, K. Baršausko 59, LT51423 Kaunas, Lithuania; algirdas.lazauskas@ktu.edu (A.L.); viktoras.grigaliunas@ktu.lt (V.G.); asta.guobiene@ktu.lt (A.G.)
[2] Department of Physics, Kaunas University of Technology, Studentu St. 50, LT51368 Kaunas, Lithuania; linas.puodziukynas@ktu.lt
* Correspondence: dalius.jucius@ktu.lt; Tel.: +370-37-313432

Received: 3 September 2020; Accepted: 5 October 2020; Published: date

Table S1. Anisotropic etching of Si (100) in 30 wt% aqueous KOH solution.

Etching parameters		Optical microscope images	Comment
**Etching temperature, °C**	**Etching time, min**		
80	**10**	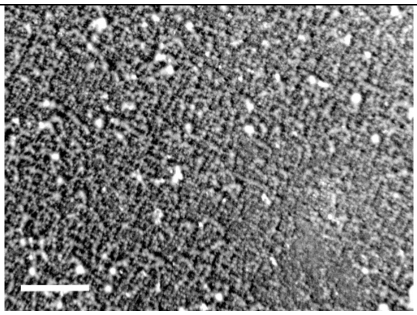 Mark size—20 μm	Inhomogeneous surface, possible over-etching
	**6**	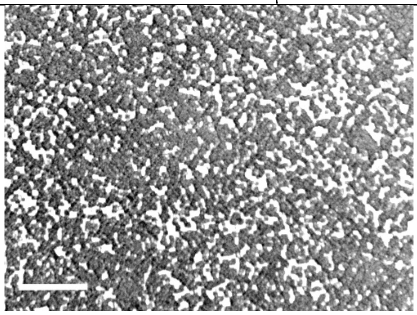 Mark size—20 μm	Inhomogeneous surface, insufficient etching
	**2**	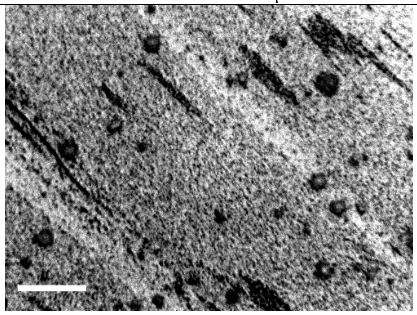 Mark size—20 μm	Insufficient etching

**65**	**10**	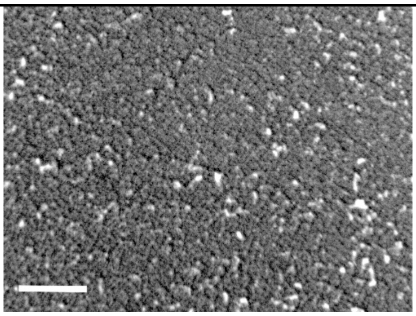 Mark size—20 μm	Homogeneous etching, black surface
	**6**	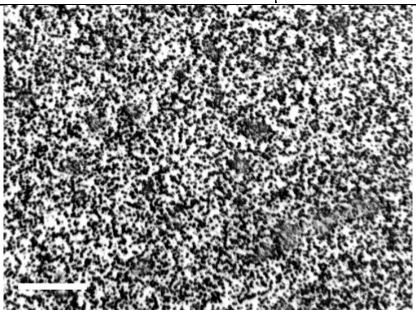 Mark size—20 μm	Homogeneous surface, insufficient etching
**50**	**10**	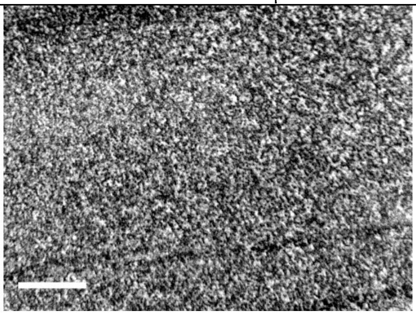 Mark size—20 μm	Inhomogeneous surface, insufficient etching

© 2020 by the authors. Submitted for possible open access publication under the terms and conditions of the Creative Commons Attribution (CC BY) license (http://creativecommons.org/licenses/by/4.0/).
